# SCORE2 cardiovascular risk prediction models in an ethnic and socioeconomic diverse population in the Netherlands: an external validation study

**DOI:** 10.1016/j.eclinm.2023.101862

**Published:** 2023-02-16

**Authors:** Janet M. Kist, Rimke C. Vos, Albert T.A. Mairuhu, Jeroen N. Struijs, Petra G. van Peet, Hedwig M.M. Vos, Hendrikus J.A. van Os, Edith D. Beishuizen, Yvo W.J. Sijpkens, Mohammad A. Faiq, Mattijs E. Numans, Rolf H.H. Groenwold

**Affiliations:** a Health Campus The Hague, Leiden University Medical Centre, The Hague, The Netherlands; b Department of Internal Medicine, HAGA Teaching Hospital, The Hague, The Netherlands; c National Institute for Public Health and the Environment, Bilthoven, The Netherlands; d National eHealth Living Lab, Leiden University Medical Centre, Leiden, The Netherlands; e Department of Internal Medicine, HMC Hospital, The Hague, The Netherlands; f Department of Clinical Epidemiology, Leiden University Medical Centre, Leiden, The Netherlands; g Department of Biomedical Data Science, Leiden University Medical Centre, Leiden, The Netherlands

**Keywords:** Cardiovascular disease, Health disparities, Risk factors, Socioeconomic factors, Ethnicity

## Abstract

**Background:**

Socioeconomic status and ethnicity are not explicitly incorporated as risk factors in the four SCORE2 cardiovascular disease (CVD) risk models developed for country-wide implementation across Europe (low, moderate, high and very-high model). The aim of this study was to evaluate the performance of the four SCORE2 CVD risk prediction models in an ethnic and socioeconomic diverse population in the Netherlands.

**Methods:**

The SCORE2 CVD risk models were externally validated in socioeconomic and ethnic (by country of origin) subgroups, from a population-based cohort in the Netherlands, with GP, hospital and registry data. In total 155,000 individuals, between 40 and 70 years old in the study period from 2007 to 2020 and without previous CVD or diabetes were included. Variables (age, sex, smoking status, blood pressure, cholesterol) and outcome first CVD event (stroke, myocardial infarction, CVD death) were consistent with SCORE2.

**Findings:**

6966 CVD events were observed, versus 5495 events predicted by the CVD low-risk model (intended for use in the Netherlands). Relative underprediction was similar in men and women (observed/predicted (OE-ratio), 1.3 and 1.2 in men and women, respectively). Underprediction was larger in low socioeconomic subgroups of the overall study population (OE-ratio 1.5 and 1.6 in men and women, respectively), and comparable in Dutch and the combined “other ethnicities” low socioeconomic subgroups. Underprediction in the Surinamese subgroup was largest (OE-ratio 1.9, in men and women), particularly in the low socioeconomic Surinamese subgroups (OE-ratio 2.5 and 2.1 in men and women). In the subgroups with underprediction in the low-risk model, the intermediate or high-risk SCORE2 models showed improved OE-ratios. Discrimination showed moderate performance in all subgroups and the four SCORE2 models, with C-statistics between 0.65 and 0.72, similar to the SCORE2 model development study.

**Interpretation:**

The SCORE 2 CVD risk model for low-risk countries (as the Netherlands are) was found to underpredict CVD risk, particularly in low socioeconomic and Surinamese ethnic subgroups. Including socioeconomic status and ethnicity as predictors in CVD risk models and implementing CVD risk adjustment within countries is desirable for adequate CVD risk prediction and counselling.

**Funding:**

10.13039/501100005039Leiden University Medical Centre and 10.13039/501100001717Leiden University.

**Research in context**

**Evidence before this study**

Ethnicity and socioeconomic status are associated with higher cardiovascular disease risk. Whether ethnicity and socioeconomic status should explicitly be included in cardiovascular disease risk prediction models on mainland Europe is still unclear. In preparation for this manuscript, PubMed was searched from inception until December 31, 2022, for “cardiovascular disease”, “cardiovascular prediction”, “socioeconomic status”, “ethnicity”, “SCORE2”, “social determinants of health” (which yielded 2600 studies).

**Added value of this study**

On top of traditional risk factors (age, smoking, cholesterol and blood pressure), socioeconomic status and ethnicity further distinguish individuals at higher or lower absolute risk for cardiovascular disease events. Adjusting risk prediction of high-risk subgroups to SCORE2 models intended for use in higher-risk European countries (best fitting model according the observed to expected events per subgroups) would, in our study, have led to an almost twofold increase of individuals eligible for treatment (from 10% to 17%, and from 2% to 5% in men and women, respectively).

**Implications of all the available evidence**

Appropriate risk modelling within countries taking ethnicity as well as socioeconomic status into account is necessary for adequate cardiovascular disease risk counselling. The existing information in routine health care data could service the population through research into health disparities to help improve health equity.

## Introduction

Cardiovascular disease (CVD) is the most common cause of death in Europe, causing 45% of all deaths.^1^^,^^2^ After decades of decreasing numbers in Europe, CVD deaths are expected to rise again due to an ageing population and unhealthy lifestyles.^2^^,^^3^

To distinguish seemingly healthy persons in Europe at low and high CVD risk, the SCORE2 was recently developed to predict the 10-year cardiovascular risk of fatal and non-fatal cardiovascular events.^4^ SCORE2 is based on pooled large prospective cohorts of almost 700,000 patients and externally validated in over one million individuals.^4^ The SCORE2 risk models are adjusted to background country-level CVD risk, on the basis of standardised CVD mortality rates, to four country-wide CVD risk models (low, moderate, high and very-high).

SCORE2 models are incorporated in the 2021 ESC Guidelines on cardiovascular disease prevention in clinical practice, which provides preventive recommendations for populations (e.g., health policies) and the individual.^5^ On the individual level, the guideline provides a stepwise approach, with lifestyle and treatment recommendations based on SCORE2 risk prediction, comorbidities, risk modifiers (e.g., family history, ethnicity, stress factors), lifetime benefit and patient preferences.^5^

The European SCORE2 prediction models are based on traditional risk factors for CVD (age, sex, smoking status, diabetes, blood pressure and cholesterol).^4^ However, studies showed large differences in CVD in socioeconomic and ethnic subgroups of the population.^6^, ^7^, ^8^, ^9^, ^10^, ^11^, ^12^, ^13^, ^14^ The differences in CVD death found in socioeconomic and ethnic subgroups within European countries, are of similar magnitude compared with the differences in CVD death between Eastern and Western European countries.^11^^,^^14^ These differences might urge targeted absolute risk adjustment for specified subgroups within countries on top of traditional risk factors. The observed differences, however, could still be the result of differences in traditional risk factors (such as diabetes, smoking and hypertension), driving the higher observed risks in these subgroups.^5^^,^^10^^,^^15^, ^16^, ^17^, ^18^

In Europe, ASSIGN was the first to incorporate socioeconomic status in their CVD risk model for the Scottish population, and the QRISK CVD risk model has incorporated socioeconomic status as well as ethnicity for use in the UK population.^19^^,^^20^ For the other European countries, it is still unclear whether ethnicity and socioeconomic status need to be incorporated in CVD risk models.^5^^,^^21^ Specifically, the SCORE2 prediction models have not been externally validated in combined ethnic and socioeconomic subgroups yet.^5^ The primary aim of this study was to evaluate the performance of the SCORE2 prediction models in the population and in different ethnic and socioeconomic subgroups.

## Methods

This external validation study was reported according to the Transparent reporting of a multivariable prediction model for individual prognosis or diagnosis statement (TRIPOD, Appendix A).^22^

### Design, population and study period

This was a prospective, dynamic, population-based study, from the novel routine health care database of the Extramural LUMC Academic Network (ELAN). For this study data from general practitioners (GPs) and hospitals of the region of The Hague in the Netherlands were linked with medical and registry data from Statistics Netherlands using unique pseudonymised identifiers for households and individuals.^23^^,^^24^ Individuals were included if they were registered between 2007 and 2020 at a GP participating in the ELAN database for at least 6 months. We excluded individuals with a history of diabetes or CVD before cohort entry, defined as non-fatal stroke or myocardial infarction, as well as individuals using statin and diabetes medication before cohort entry (flowchart in Appendix B).

Cohort entry was defined as the date a person was registered with a participating GP, start of 2007 and age between (turning) 40 and 70 years. Follow-up time in person years was calculated from cohort entry until development of an event (CVD death, first CVD event), attaining age 80 (70 plus 10-year follow-up), end of study period (July 2020) or deregistration with a participating GP practice, whichever came first.

### Outcome and competing event

The main outcome of this study was similar to SCORE2, a combined endpoint of CVD death and first CVD event after cohort entry (stroke or myocardial infarction). Competing events were non-CVD deaths. Deaths were derived from the death registry of Statistics Netherlands, coded according to the International Classification of Diseases and Related Health Problems 10th version (ICD-10). ICD-10 death codes have been found to be reliable in 98% of the cases.^25^ CVD events were extracted from GP files, coded according to the International Classification of Primary Care (ICPC), consistent with SCORE2 ICD-10 codes. The complete list of used CVD codes can be found in Appendix C.

### Predictors of cardiovascular events

For the validation of the SCORE2 prediction model, the variables age, sex, smoking, non-HDL-cholesterol and systolic blood pressure were used. Age and sex were derived at cohort entry. Individual data on smoking, blood pressure and non-HDL cholesterol were derived from medical records (GP when available, otherwise from hospital data, value before or nearest to cohort entry). Medical data from GPs and hospital were linked with individual data from Statistics Netherlands, on deaths, sex, age, ethnicity (country of origin) and socioeconomic status (disposable household income, derived from Dutch tax register). Additional information on smoking status was derived from text mining (string matching) on free text information within the GP data (details in Appendix D). Ethnicity of individuals was based on the country of origin (classification as used by Statistics Netherlands).^24^

For this study, disposable household income which represents “the net amount a household can spend on an annual basis, adjusted for any differences in household size and composition” was used as a proxy for socioeconomic status.^24^ The information on disposable household level provided by Statistics Netherlands consisted of percentiles of disposable household level as compared to the general population of the Netherlands.^24^ The quintile cut-points for 2014 were €16,000, €21,100, €26,800 and €34,700.

### Statistical analysis

Analyses were performed in the overall population by sex and stratified by ethnicity and socioeconomic status. For external validation, the original SCORE2 Fine and Gray model formulas, syntax, intercept and regression-coefficients were provided by the SCORE2 research team.^4^ After 10 years of follow-up, the risk predictions for the low, moderate, high and very high-risk models were calculated. On sample size considerations, external validation was performed on subgroups with at least 200 CVD events (minimal numbers for an appropriate external validation).^26^

Calibration was assessed with observed/mean expected probability (OE-ratio) at timepoint 10 years and with calibration plots in deciles of the population by predicted risk. Discrimination was assessed using Harrell's C-statistic.

Pre-processing and analyses of the data were performed using R Statistical Computing (version 4.2.1). Data were analysed on the secure data infrastructure of Statistics Netherlands.

On missing data, when information on a binary variable was not reported, for example a CVD diagnosis, it was assumed that particular variable was absent. On variables, percentages of missingness were reported. For disposable household income, blood pressure and cholesterol we assumed these were missing at random and were imputed using multiple imputation (variables in the imputation model: age, blood pressure, cholesterol, fasting glucoses, eGFR, smoking status, disposable household income, ethnicity, follow-up time and CVD events). Five imputed datasets were generated using the MICE algorithm in R, the convergence of imputed values was assessed in 40 imputations, and results were combined with Rubin's rule.^27^

Several sensitivity analyses were performed. First, to assess differences due to missingness, performance analyses were repeated in part of the population with at least 1 measurement. Second, to assess spatial and socioeconomic trends we analysed part of the population from a smaller, more urban area of The Hague with and without imputation of missing socioeconomic status. Third, performance variation in age groups (40–50 and 50–70 years) was assessed, because SCORE2 treatment thresholds are different under and above 50 years of age (2.5 and 7.5% versus 5 and 10% predicted probability). Fourth, to assess validity of the CVD event outcome, we compared CVD events (ICPC coded) with start of the combination of statins and antithrombotic medication in the same individual.

### Ethics

Routinely collected data were anonymized through a trusted third party and Statistics Netherlands to prevent identification of individuals by researchers. In accordance to Dutch legislation, GPs and hospitals informed individuals about use of their anonymized data for research purposes and individuals could withdraw via an informed opt-out procedure and informed consent from individuals in the study was waived and not obtained. For this waiver, the appropriate approval that the study is not subject to the Medical Examination Act was granted, after evaluation of the research protocol by the authority of the area (Medical Ethical Committee LUMC Leiden, under reference number G18.070).

### Role of the funding source

The ELAN Vascular study is supported by the Department of Public Health & Primary care and the Board of Directors of the Leiden University Medical Centre and by the Leiden University. The funding sources had no role in study design, collection, analysis, interpretation of the data, in the writing of the report, or the decision to submit the paper for publication.

## Results

Data of 74,880 men and 80,133 women from the region of The Hague were included from January 1st, 2007 to July 1st, 2020 (based on the in- and exclusion criteria, from 537,000 individuals in the ELAN Datawarehouse).

In men 4251 CVD events and in women 2715 CVD events occurred. The mean age was 48 years (SD 9). Compared to the income boundaries of the overall Dutch population, our population showed a different distribution, with lower numbers of individuals in the lower disposable household income quintiles. For men, 64% were of Dutch origin, 6% of Surinamese, for women, 62% were Dutch and 7% Surinamese (Table 1). With lower numbers, the other ethnicities were combined as one group (31% of men and women). Mean systolic blood pressure was 136 mmHg (SD 22, 31% missing), mean total cholesterol was 5.5 mmol/l (SD 1.1, 35% missing), and mean HDL cholesterol 1.4 mmol/L (SD 0.4, 36% missing). The median follow-up time was 9.9 years (interquartile range, IQR 5.7–12.8).

**Table 1:** Baseline characteristics routine health care cohort, 2007–2020, 40–70 years of age.

	Men	CVD events	Women	CVD events	Missing	*SCORE2 derivation cohort*
	n (%), mean (SD)	n	n (%), mean (SD)	n	%	*n (%), mean (SD)*
Total	74,880		80.133			*677.684*
Age at cohort entry (years)	48.1 (8.6)		48.3 (8.8)			*57 (9)*
Systolic blood pressure (mmHg)	138 (21)		134 (22)		31	*136 (19)*
Total cholesterol (mmol/l)	5.5 (1.1)		5.5 (1.1)		35	*5.8 (1.1)*
HDL cholesterol (mmol/l)	1.2 (0.3)		1.5 (0.4)		36	*1.4 (0.4)*
Smoking	24,978 (33.4)		25,751 (32.1)		a	*101,211 (15)*
Follow-up (person-years, median, IQR)	9.8 (5.6–12.6)		10.1 (5.8–12.8)			*10.7 (5.0–18.6)*
Events						
CVD events	4251 (5.7)		2715 (3.4)			*30,121 (4.4)*
Non-CVD death	2100 (2.8)		1918 (2.4)			*33,809 (5.0)*
Subgroups						
Socioeconomic status					6	
1st (lowest)	11,121 (15.9)	653	13,487 (16.8)	555		
2nd	8889 (12.7)	555	11,110 (13.9)	489		
3rd	12,298 (17.6)	704	13,857 (17.3)	473		
4th	16,274 (23.3)	968	16,547 (20.6)	544		
5th (highest)	21,411 (30.6)	1196	20,946 (26.1)	555		
Ethnicity (by country of origin)						
Dutch	47,901 (64.0)	2897	49,776 (62.1)	1893		
Surinamese	4143 (5.5)	300	5492 (6.9)	237		
Other ethnicities:						
Antilleans	1445 (1.9)	66	1675 (2.1)	48		
Brittons	932 (1.2)	29	802 (1.0)	22		
Germans	1071 (1.4)	97	1216 (1.5)	76		
Indonesian	4122 (5.5)	276	4466 (5.6)	141		
Moroccan	2256 (3.0)	81	1980 (2.5)	22		
Turkish	2246 (3.0)	118	1970 (2.5)	45		
Middle & Eastern Europeans	1006 (1.3)	33	1632 (2.0)	29		
Other countries	9758 (13.0)	354	11,124 (13.9)	202		

SD, standard deviation.

CVD events, cardiovascular disease events, combination of CVD death and first CVD event.

CVD death, ICD-10 I10-I25, R96, I46, I47-I51, I61-I65 (except I62.0), G45, I67-I69 (except I67.1), I70 tot I72.

CVD event, myocardial infarction and stroke (ICPC K75, K90, except K90.1).

Socioeconomic status, by disposable household income, in quintiles of the population of the Netherlands.

a Missing in 50% of cases, when missing non-smoking was assumed.

### Performance measures SCORE2

#### Performance: Total population

**Fig. 1:**
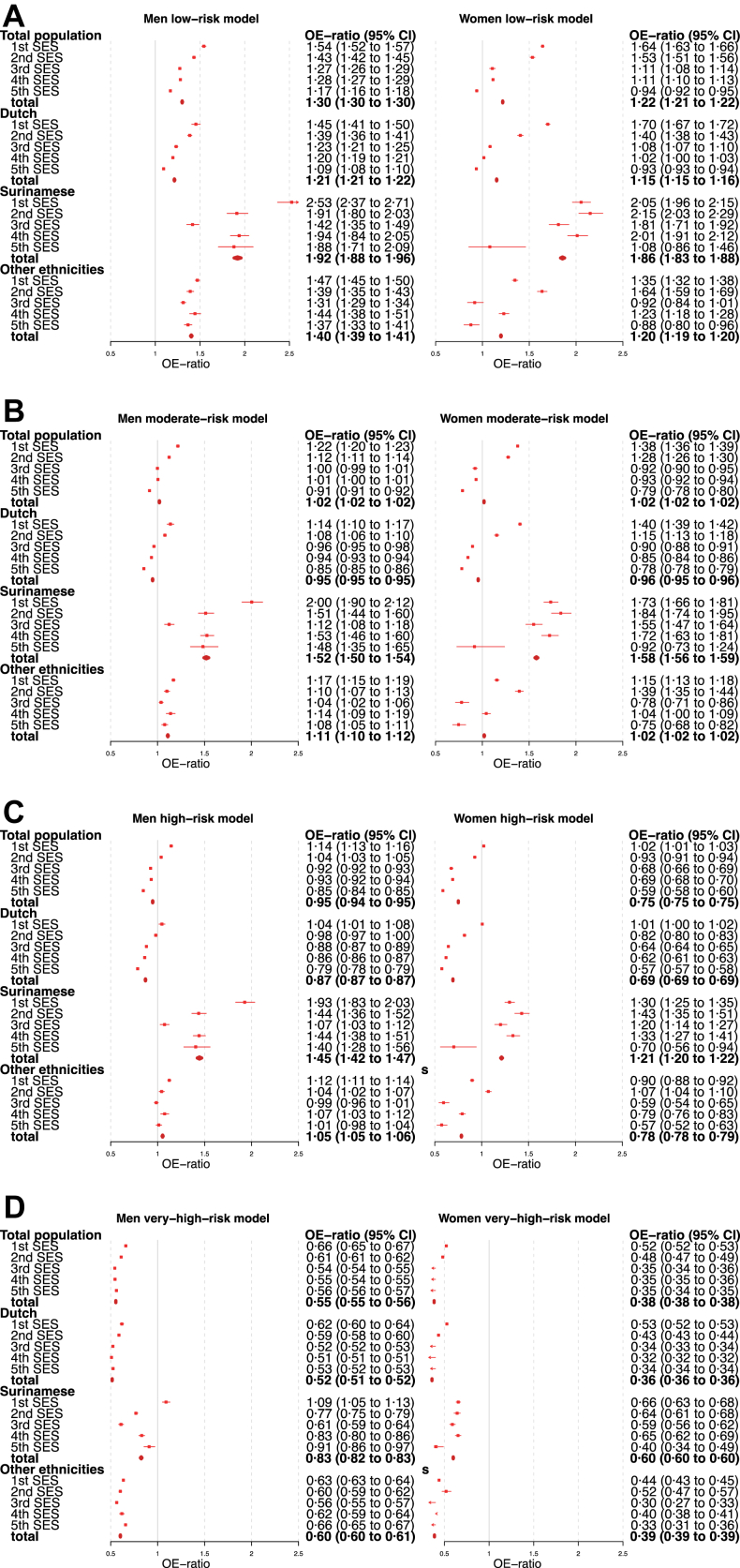
**OE-ratio external validation SCORE2 by ethnicity and socioeconomic status.** OE-ratio, observed risk/mean predicted probability (<1 overprediction, >1 underprediction), the OE-ratio is an overall assessment of the correspondence of the predicted probability compared to the actual observed risks. 1st–5th SES, socioeconomic status, by disposable household income, in quintiles of the population of the Netherlands, 1st SES is lowest, 5th SES is highest. Low-, moderate-, high- and very-high-risk model, the four SCORE2 models for different regions of Europe, the low-risk model is the intended model for the Netherlands.

At external validation of the SCORE2 low-risk model (intended for use in the Netherlands), we found that for the total population the SCORE2 low-risk model predicted 5495 CVD events in the population included in this study, whereas 6966 CVD events were observed. Calibration showed underprediction both in men and women. Calibration showed an OE-ratio (observed/mean expected ratio) of 1.30 (95% confidence interval (CI) 1.30–1.30) in men and 1.22 (95% CI 1.21–1.22) in women (Fig. 1).

The moderate-risk model showed an OE-ratio of 1.02 (95% CI 1.02–1.02) in men and women. The high-risk model and very-high-risk model (intended for use in Eastern Europe) showed overprediction in men and women for the total population, with an OE-ratio below 1 (Fig. 1). Discrimination showed moderate performance in all regional SCORE2 risk models, with a C-statistic of 0.70 (95% CI 0.69–0.71) and 0.72 (95% CI 0.71–0.73) in men and women, respectively (Table 2).

**Table 2:** Discrimination external validation SCORE2 by ethnicity and socioeconomic status.

Subgroups	Harrell's C-statistic (95% CI)	
	Men	Women
Total population		
1st SES	0.70 (0.68–0.72)	0.73 (0.71–0.75)
2nd SES	0.70 (0.68–0.72)	0.73 (0.69–0.77)
3rd SES	0.70 (0.68–0.72)	0.72 (0.70–0.74)
4th SES	0.70 (0.68–0.71)	0.70 (0.68–0.72)
5th SES	0.72 (0.70–0.73)	0.71 (0.69–0.73)
**Total**	**0.70 (0.69–0.71)**	**0.72 (0.71–0.73)**
Dutch		
1st SES	0.67 (0.64–0.70)	0.69 (0.66–0.72)
2nd SES	0.70 (0.67–0.72)	0.72 (0.68–0.75)
3rd SES	0.70 (0.68–0.72)	0.72 (0.69–0.74)
4th SES	0.69 (0.67–0.71)	0.72 (0.69–0.74)
5th SES	0.72 (0.71–0.74)	0.71 (0.68–0.73)
**Total**	**0.70 (0.69–0.71)**	**0.72 (0.70–0.73)**
Surinamese		
1st SES	0.63 (0.57–0.70)	0.71 (0.65–0.77)
2nd SES	0.63 (0.54–0.71)	0.74 (0.60–0.88)
3rd SES	0.64 (0.55–0.73)	0.69 (0.62–0.76)
4th SES	0.68 (0.61–0.75)	0.63 (0.55–0.71)
5th SES	0.69 (0.62–0.76)	0.71 (0.63–0.80)
**Total**	**0.65 (0.62–0.69)**	**0.70 (0.67–0.73)**
Other ethnicities		
1st SES	0.74 (0.71–0.77)	0.76 (0.72–0.80)
2nd SES	0.71 (0.66–0.75)	0.75 (0.68–0.82)
3rd SES	0.73 (0.68–0.77)	0.74 (0.69–0.79)
4th SES	0.72 (0.68–0.76)	0.69 (0.63–0.75)
5th SES	0.71 (0.68–0.75)	0.72 (0.65–0.78)
** Total**	**0.72 (0.71–0.74)**	**0.74 (0.72–0.76)**

Harrell's C-statistic, displays whether the model accurately discriminates individuals with the event from individuals without the event (when 1, discrimination of a model is perfect, when 0.5, the model does not discriminate between individuals with and without the event).

Ethnicity, by country of birth.

1st–5th SES, socioeconomic status, by disposable household income, in quintiles of the population of the Netherlands, 1st SES is lowest, 5th SES is highest.

#### Performance: Socioeconomic subgroups

In general, at calibration in socioeconomic subgroups for the total population the SCORE2 models showed more extreme underprediction in subgroups at lower socioeconomic status. The OE-ratios of the low-risk model, ranged in the total population in lowest to highest socioeconomic quintile from 1.54 (95% CI 1.52–1.57) to 1.17 (95% CI 1.16–1.18) in men, and from 1.64 (95% CI 1.63–1.66) to 0.94 (95% CI 0.92–0.95) in women. Calibration plots by socioeconomic subgroups showed similar results (Appendix E).

Discrimination showed moderate performance in all four risk models and across subgroups. For the low-risk model in socioeconomic subgroups, C-statistics ranged from 0.70 to 0.72, and 0.70 to 0.73 in men and women respectively (Table 2).

#### Performance: Ethnic subgroups

**Fig. 2:**
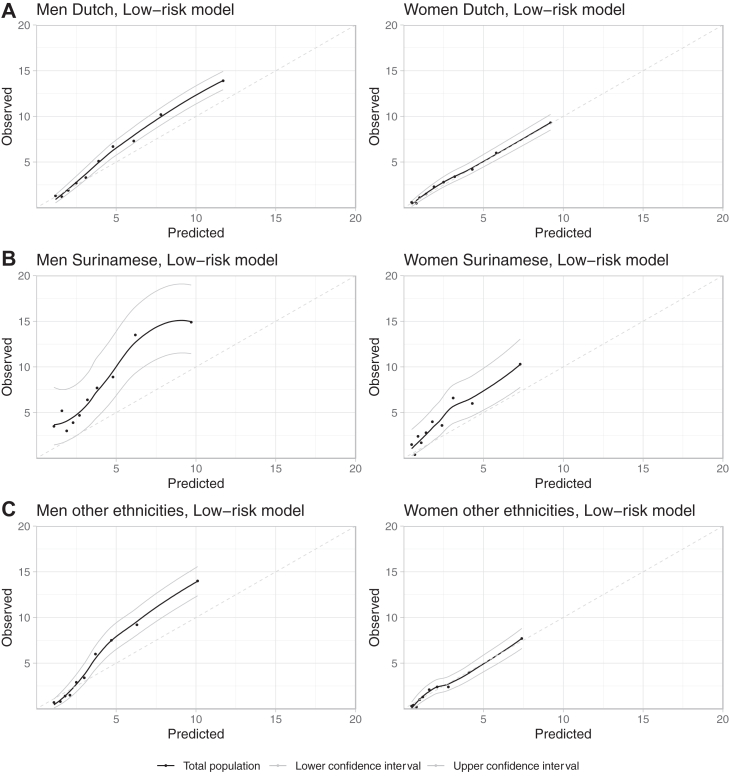
**Calibration plots, SCORE2 low-risk model by ethnicity (LOESS plotted).** Calibration plots, asses model fit by graphically comparing predicted probability to the proportion of observed events in decile groups of the population. Loess, locally estimated scatterplot smoothing.

Performance was assessed in the Dutch, Surinamese and other ethnicity subgroups. Calibration in the Dutch and other ethnicity subgroups showed underprediction (OE-ratio low-risk model Dutch and 1.21 (95% CI 1.21–1.22) and 1.15 (95% CI 1.14–1.16) in men and women, respectively). In “other ethnicities” 1.40 (95% CI 1.35–1.44) and 1.20 (95% CI 1.02–1.38) in men and women, respectively (Fig. 1, calibration plots Fig. 2). In Surinamese, calibration showed underprediction to a greater distance (OE-ratio in the low-risk model was 1.92 (95% CI 1.89–1.95) in men and 1.86 (95% CI 1.81–1.92) in women (Fig. 1), calibration plots Fig. 2).

For “other ethnicities” men the high-risk model 1.05 (95% CI 1.05–1.06) was more nearing one in the OE-ratio, while for Surinamese men and women, the high-risk model still had an OE-ratio of 1.45 (95% CI 1.42–1.47) and 1.21 (95% CI 1.20–1.22), respectively (Fig. 1).

Discrimination showed moderate performance in all regional SCORE2 risk models, with a C-statistic in Dutch of 0.70 (95% CI 0.69–0.71) and 0.72 (95% CI 0.71–0.73), in “other ethnicities” 0.72 (95% CI 0.71–0.74) and 0.74 (95% CI 0.72–0.76) and in Surinamese, 0.65 (95% CI 0.62–0.69) and 0.70 (95% CI 0.67–0.73), in men and women respectively (Table 2).

#### Performance: Combined socioeconomic and ethnic subgroups

At calibration in the combined socioeconomic and ethnic subgroups, the SCORE2 models showed more extreme underprediction in subgroups at lower socioeconomic status.

In combined subgroups of men, OE-ratios were highest in the Surinamese subgroups, from 2.53 (95% CI 2.37–2.71) to 1.88 (95% CI 1.71–2.09) and lowest in the Dutch subgroups, from 1.45 (95% CI 1.41–1.50) to 1.09 (95% CI 1.08–1.10). In women, the Surinamese had the highest OE-ratios, from 2.05 (95% CI 1.96–2.15) to 1.08 (95% CI 0.86–1.46), and the lowest were the “other ethnicities” subgroup, from 1.35 (95% CI 1.32–1.38) to 0.88 (95% CI 0.80–1.35) (Fig. 1).

Discrimination in the combined socioeconomic and ethnic subgroups showed moderate performance in all four risk models (C-statistics ranged from 0.63 to 0.74, and 0.63 to 0.76 in men and women respectively, Table 2).

### Sensitivity analyses

First, analyses of the subjects in whom at least one laboratory measurement was made showed slightly lower or slightly higher observed risks. Second, in the analyses in the smaller more urban area of the city of The Hague, the proportion of individuals in the lowest quintile of household income was higher, and missingness was approximately 5% higher, but calibration and discrimination of the smaller area as well as with and without information on household income, showed similar results in all subgroups. Third, when we stratified on age between forty and fifty and over fifty, calibration showed more underprediction in men 50 and above and in women between 40 and 50 years of age, discrimination was slightly lower in these age subgroups. Fourth, 93% of individuals with a CVD event (first myocardial infarction or stroke) started with the combination of statin and antithrombotic medication.

## Discussion

This study aimed to externally validate the SCORE2 CVD risk prediction models with a focus on its performance in socioeconomic and ethnic subgroups. In the general study population, the performance of the SCORE2 low-risk model (intended for use in the Netherlands) showed underprediction in men and slight underprediction in women. However, in terms of calibration the performance of the SCORE2 low-risk model was particularly poor in low socioeconomic subgroups and in Surinamese subgroups. The moderate or high-risk SCORE2 model appeared to be a much better predictive model for these subgroups.

Our study also showed that information available in contemporary routine health care data combined with national registry databases in the Netherlands can be utilized for research into health disparities, ethnicity and socioeconomic status.

While much evidence exists on the relation between socioeconomic status, ethnicity and the risk for CVD, the SCORE2 CVD risk prediction models do not take these factors into account explicitly.^4^^,^^5^ The observed higher CVD risk in lower socioeconomic subgroups is consistent with literature since the 1970s.^6^^,^^9^ Evidence on the combined effect on absolute risk of socioeconomic status and traditional risk factors was inconclusive for the European main land population.^5^ In CVD risk models in Scotland and the UK, ASSIGN and QRISK include socioeconomic status since 2007 in their risk models, with socioeconomic area codes (Scottish Index of Multiple Deprivation score SIMD and Townsend score, respectively).^19^^,^^28^ SIMD is based on income, employment, education, health, access to services, crime and housing.^19^ The Townsend score is based on unemployment, car ownership, home ownership and household overcrowding.^29^ Individual information on socioeconomic status is however preferable and income is a valid measure for the association between socioeconomic status and CVD.^30^ One study assessed the performance of SCORE2 in Scotland, and found comparable higher CVD OE-ratios in lowest socioeconomic subgroups.^31^ Also, in the performed sensitivity analyses in suburban and urban areas, low socioeconomic status subgroups showed a comparable increased absolute risk. With 74% of the population living in a suburban or urban area in the Netherlands, this could indicate a generalizable risk increase for low socioeconomic subgroups in the general population.^32^

The ESC guideline recommends to use absolute risk multipliers for ethnicities (factor 1.3 for Indian, 1.7 for Pakistan and 0.85 for African Caribbean descendance).^5^ However, these multipliers were specifically developed for ethnic subgroups in the UK, and might not be applicable for ethnic subgroups in other European regions.^5^^,^^21^ The CVD risk among Surinamese in our The Hague region (76% of South Asian and 13% of African Caribbean descendance) was 1.9 times higher than the predicted CVD probabilities (men and women), which is comparable with the multiplier found in Pakistan descendants in the UK, but is higher than the UK multiplier for individuals of Indian and African Caribbean descent.^32^

In the Dutch and the combined “other ethnicities” subgroups we found comparable higher risks for CVD events in lower socioeconomic subgroups. The Surinamese subgroups (76% South Asian) showed higher observed risks for CVD, with highest OE ratios in lowest socioeconomic Surinamese. It has been hypothesized that higher CVD risk in individuals of low socioeconomic status or of South Asian decent is due to complex intermixing factors, e.g., (epi)genetical, stress and lifestyle differences.^15^^,^^18^^,^^33^, ^34^, ^35^, ^36^ These partly known complex intermixing factors probably contribute as to why traditional CVD risk factors combined with socioeconomic status or ethnicity do not account for an appropriate risk prediction.^7^^,^^35^, ^36^ Given that the observed risk distinctions in the socioeconomic and ethnic subgroups in our study go beyond the risk predicted by traditional risk factors and are as large as those encountered between western and eastern Europe populations, individualized absolute CVD risk adjustment based on both ethnicity and socioeconomic status within countries is warranted.

Overall, the men and women in our population showed a higher observed risk for CVD events compared to expected probability based on the SCORE2 models. Given the individuals in the study population were generally younger compared to the development cohort, this is an unexpected result. This study was performed in a contemporary population. Possibly this unexpected higher CVD risk might be due to the general increase of unhealthier lifestyles in the Netherlands, with for example an increase of obesity from 42% in 2007 to 50% in 2020.^37^

This study has several limitations. First, the missingness in our cohort in smoking, cholesterol and blood pressure is considerable. Even though the percentage of missingness was similar to similar cohorts (20–50% missing values versus e.g., 15–60% missing values across different variables in the QRISK derivation study), but still QRISK yields robust external validation results.^21^ We performed several sensitivity analyses in parts of the cohort with 20–50% missingness, which yielded similar results in calibration. We expect that the missingness in this CVD calibration has not affected our performance measures. Second, the study population was not large enough to analyse calibration in more ethnic subgroups. However, we were able to stratify the two largest ethnicity subgroups, and combined the other ethnic subgroups. Third, country of origin was the only available source as a proxy for ethnicity. Information on (self-perceived) ethnicity of the Surinamese subgroup would have been preferable and could have led to more specific results on individuals of South Asian or African Caribbean descendance.

Our study also has strengths. First this study is, to our knowledge, the first to assess the predictive performance of the SCORE2 CVD risk prediction model in a contemporary population stratified by socioeconomic status and ethnicity. The combination of routine health care data from GPs and hospitals, combined with Statistics Netherlands data is unique and the only database yet in the Netherlands where data from these 3 sources are combined for research on health inequities/inequalities. Furthermore, traditional cohorts usually have an underrepresentation of individuals with a low socioeconomic background or different ethnicities, whereas routine health care databases are inclusive of all individuals in countries within a universal health care system.

The developed 4 SCORE2 risk prediction models would probably fit the majority of the socioeconomic and ethnic diverse population of Europe. Currently, national CVD mortality data guides the choice for country wide implementation of one of the SCORE2 models. As a short-term future research direction, absolute CVD risk differences in national mortality data in (combined) socioeconomic and ethnic subgroups could be estimated. Based on these CVD risk differences, ESC/SCORE2 could consider to advise implementation of the SCORE2 low, moderate-, high- or very-high-risk models for specific socioeconomic and ethnic subgroups within countries.

In the long-term, the expanding development of population-based research cohorts in Europe, combined with traditional cohorts, could be beneficial for further finetuning risk prediction to existing health disparities in European populations. For population-based CVD prediction research we recommend including information on self-perceived ethnicity, and additional risk elevating predictors, such as body mass index, pregnancy related risk factors and family history of CVD.

Shifting toward applying higher CVD risk models within countries in high-risk subgroups, would in our population have led to an increase of the population meeting treatment thresholds from 10% to 17%, and from 2% to 5%, in men and women, respectively.

In the Netherlands, as a proxy for low socioeconomic status, eligibility for housing rental benefits or financial health care benefits could be used.

In conclusion, our study showed that the SCORE 2 CVD risk model for low-risk countries (as the Netherlands are) was found to underpredict CVD risk, particularly in low socioeconomic and Surinamese ethnic subgroups. Including socioeconomic status and ethnicity as predictors in CVD risk models and implementing CVD risk adjustment within countries is desirable for adequate CVD risk prediction and counseling.

## Contributors

J.M.K., M.E.N., R.H.H.G., J.N.S., R.C.V., P.G.v.P., A.T.A.M., H.M.M.V., E.D.B., H.J.A.v.O. and Y.W.J.S. contributed to the conception and design of the work. JM.K, M.E.N., J.N.S., A.T.A.M., H.M.M.V., E.D.B. and Y.W.J.S. contributed to the acquisition of the data. The data was analysed, and the underlying data was accessed and verified by J.M.K., M.A.F. and R.H.H.G. J.M.K. drafted the manuscript and figures. All authors contributed to the interpretation of the data analyses, and critically revised the manuscript. All authors read and approved the final version.

## Data sharing statement

The data used for this study is part of a larger study on health equity in the city of The Hague (*ELAN Datawarehouse*, sub study ELAN Vascular). Data used for this study is prohibited from sharing, although, requests for collaborations on the research project ELAN Vascular, can be addressed to the Health Campus of the Leiden University Medical Centre (https://healthcampusdenhaag.nl/). The research protocol, coding and data dictionary used for this study is available on request via the author (J.M.Kist@lumc.nl).

## Declaration of interests

All authors declare no competing interests.
