# Supplementary material for: SCORE2 cardiovascular risk prediction models in an ethnic and socioeconomic diverse population in the Netherlands: an external validation study
Source: eClinicalMedicine. 2023 Feb 16;57:101862. doi: 10.1016/j.eclinm.2023.101862 (PMC9971516; doi:10.1016/j.eclinm.2023.101862)
Supplement: Appendix A–E [file mmc1.docx]

**Appendix A. Transparent reporting of a multivariable prediction model for individual prognosis or diagnosis statement (TRIPOD)**

| **Section/Topic** | **Item** | **Checklist Item** | **Page** |
| --- | --- | --- | --- |
| **Title and abstract** | | | |
| Title | 1 | Identify the study as developing and/or validating a multivariable prediction model, the target population, and the outcome to be predicted. | 1 |
| Abstract | 2 | Provide a summary of objectives, study design, setting, participants, sample size, predictors, outcome, statistical analysis, results, and conclusions. | 2 |
| **Introduction** | | | |
| Background and objectives | 3a | Explain the medical context (including whether diagnostic or prognostic) and rationale for developing or validating the multivariable prediction model, including references to existing models. | 3,4 |
|  | 3b | Specify the objectives, including whether the study describes the development or validation of the model or both. | 4 |
| **Methods** | | | |
| Source of data | 4a | Describe the study design or source of data (e.g., randomized trial, cohort, or registry data), separately for the development and validation data sets, if applicable. | 5 |
|  | 4b | Specify the key study dates, including start of accrual; end of accrual; and, if applicable, end of follow-up. | 5 |
| Participants | 5a | Specify key elements of the study setting (e.g., primary care, secondary care, general population) including number and location of centres. | 5 |
|  | 5b | Describe eligibility criteria for participants. | 5 |
|  | 5c | Give details of treatments received, if relevant. | Not applicable |
| Outcome | 6a | Clearly define the outcome that is predicted by the prediction model, including how and when assessed. | 5,6 |
|  | 6b | Report any actions to blind assessment of the outcome to be predicted. | Not applicable |
| Predictors | 7a | Clearly define all predictors used in developing or validating the multivariable prediction model, including how and when they were measured. | 6 |
|  | 7b | Report any actions to blind assessment of predictors for the outcome and other predictors. | Not applicable |
| Sample size | 8 | Explain how the study size was arrived at. | 5 |
| Missing data | 9 | Describe how missing data were handled (e.g., complete-case analysis, single imputation, multiple imputation) with details of any imputation method. | 6,7 |
| Statistical analysis methods | 10c | For validation, describe how the predictions were calculated. | 7 |
|  | 10d | Specify all measures used to assess model performance and, if relevant, to compare multiple models. | 7 |
|  | 10e | Describe any model updating (e.g., recalibration) arising from the validation, if done. | Not applicable |
| Risk groups | 11 | Provide details on how risk groups were created, if done. | 6 |
| Development vs. validation | 12 | For validation, identify any differences from the development data in setting, eligibility criteria, outcome, and predictors. | 6 |
| **Results** | | | |
| Participants | 13a | Describe the flow of participants through the study, including the number of participants with and without the outcome and, if applicable, a summary of the follow-up time. A diagram may be helpful. | 8, Table 1, Appendix B |
|  | 13b | Describe the characteristics of the participants (basic demographics, clinical features, available predictors), including the number of participants with missing data for predictors and outcome. | 8, Table 1 |
|  | 13c | For validation, show a comparison with the development data of the distribution of important variables (demographics, predictors and outcome). | Table 1 |
| Model performance | 16 | Report performance measures (with CIs) for the prediction model. | 8, 9 |
| Model-updating | 17 | If done, report the results from any model updating (i.e., model specification, model performance). | Not applicable |
| **Discussion** | | | |
| Limitations | 18 | Discuss any limitations of the study (such as nonrepresentative sample, few events per predictor, missing data). | 12,13 |
| Interpretation | 19a | For validation, discuss the results with reference to performance in the development data, and any other validation data. | 11,12 |
|  | 19b | Give an overall interpretation of the results, considering objectives, limitations, results from similar studies, and other relevant evidence. | 11,12,13 |
| Implications | 20 | Discuss the potential clinical use of the model and implications for future research. | 13,14 |
| **Other information** | | | |
| Supplementary information | 21 | Provide information about the availability of supplementary resources, such as study protocol, Web calculator, and data sets. | 14,15 |
| Funding | 22 | Give the source of funding and the role of the funders for the present study. | 1, 15 |

**Appendix B. Flowchart**

**Appendix C. CVD codes**

|  | ICD10  *(from SCORE2)* | ICPC |
| --- | --- | --- |
| **Non-fatal cardiovascular disease** | | |
| Non-fatal myocardial infarction | I21-I23 | K75 |
| Non-fatal stroke | I60-69 | K90 |
|  |  |  |
| ***Excluded from the non-fatal stroke endpoint:*** | | |
| Subarachnoid haemorrhage | I60 |  |
| Subdural haemorrhage | I62 |  |
| Cerebral aneurysm | I67.1 |  |
| Cerebral arteritis | I68.2 |  |
| Moya Moya | I67.5 |  |

| **CVD death (from SCORE and SCORE2)** | **ICD-10 (also sub diagnoses)** |
| --- | --- |
| Hypertension related diseases | I10-I16 |
| Angina Pectoris | I20 |
| Acute myocardial infarct | I21 |
| Subsequent myocardial infarct | I22 |
| Complications after myocardial infarct | I23 |
| Other ischemic cardiac disease | I24 |
| Chronic ischemic cardiac disease | I25 |
| Cardiac arrest | I46 |
| Cardiac arrythmias | I47-I50 |
| Complications and ill-defined descriptions of heart disease | I51 |
| Transient ischemic attack - TIA | G45 |
| Nontraumatic intracerebral haemorrhage | I61 |
| CVA | I63 |
| Occlusion and stenosis of precerebral arteries | I65 |
| Other cerebrovascular diseases (excluding I67.1) | I67-I69, I62, I64 (except I67.1) |
| Atherosclerosis | I70 |
| Aneurysm | I71-I72 |
| Other sudden death, cause unknown | R96 |

**Appendix D, Text string matching terms for smoking (in Dutch)**

SOEPCODE in ('O','S')

--select count(*) from roken.journaal

IF OBJECT_ID('tempdb..#term1') IS NOT NULL DROP TABLE #term1;

CREATE TABLE #term1 (term VARCHAR(50) NOT NULL)

INSERT #term1 VALUES ('rookt' )

INSERT #term1 VALUES ('roken+')

INSERT #term1 VALUES ('roken +')

INSERT #term1 VALUES ('roken ++' )

INSERT #term1 VALUES ('sigaretten per dag' )

INSERT #term1 VALUES ('roken ja' )

INSERT #term1 VALUES ('pack(-)year(s)' )

INSERT #term1 VALUES ('py' )

INSERT #term1 VALUES ('pj' )

INSERT #term1 VALUES ('stoppen roken lukt niet' )

INSERT #term1 VALUES ('r+')

INSERT #term1 VALUES ('hulp bij stoppen met roken')

INSERT #term1 VALUES ('champix')

INSERT #term1 VALUES ('varenicline')

INSERT #term1 VALUES ('rokn')

INSERT #term1 VALUES ('rkn')

INSERT #term1 VALUES ('Minder roken lukt niet')

INSERT #term1 VALUES ('Roken: ja')

INSERT #term1 VALUES ('ROKENNHG:Ja')

INSERT #term1 VALUES ('weer gaan roken')

INSERT #term1 VALUES ('sig per dag')

INSERT #term1 VALUES ('begonnen met roken')

INSERT #term1 VALUES ('roken++')

INSERT #term1 VALUES ('niet gestopt met roken')

IF OBJECT_ID('tempdb..#term2') IS NOT NULL DROP TABLE #term2;

CREATE TABLE #term2 (term VARCHAR(50) NOT NULL)

INSERT #term2 VALUES ('roken gestopt' )

INSERT #term2 VALUES ('roken voorheen')

INSERT #term2 VALUES ('roken: voorheen' )

INSERT #term2 VALUES ('roken: vroeger' )

INSERT #term2 VALUES ('rookte vroeger' )

INSERT #term2 VALUES ('vroeger gerookt' )

INSERT #term2 VALUES ('gestopt met roken' )

INSERT #term2 VALUES ('ROKENNHG:voorh' )

INSERT #term2 VALUES ('met roken gestopt' )

IF OBJECT_ID('tempdb..#term3') IS NOT NULL DROP TABLE #term3;

CREATE TABLE #term3 (term VARCHAR(50) NOT NULL)

INSERT #term3 VALUES ('roken nee' )

INSERT #term3 VALUES ('r-')

INSERT #term3 VALUES ('nooit gerookt' )

INSERT #term3 VALUES ('rookt niet' )

INSERT #term3 VALUES ('roken-' )

INSERT #term3 VALUES ('roken: nee' )

INSERT #term3 VALUES ('Roken: nooit' )

INSERT #term3 VALUES ('ROKENNHG:NOOIT' )

Complete SQL syntax available through the corresponding author

**Appendix E. Calibration plots SCORE2 low-risk model by socioeconomic status**
